# Movement Between Suffering and Health—Experience of Health During Long‐Term Illness

**DOI:** 10.1111/scs.70069

**Published:** 2025-06-23

**Authors:** Monika Koskinen, Camilla A. Koskinen

**Affiliations:** ^1^ Department of Health Sciences, Faculty of Education and Welfare Studies Åbo Akademi University Vaasa Finland; ^2^ Faculty of Health Sciences, Department of Caring and Ethics University of Stavanger Stavanger Norway

**Keywords:** caring science, health, in‐depth interviews, long‐term illness, suffering, thematic analysis

## Abstract

**Background:**

This study takes as its starting‐point the assumption highlighted in previous research and theory that human beings can experience health despite chronic or severe illness. An individual's perceptions of health often change when being is faced with long‐term illness, the meaning of health takes new dimensions and sets health in motion. Previous studies have focused on health related to specific diseases grounded in coping strategies or experiences of meaning in life.

**Aim:**

This study aims to gain a deeper understanding of health, what it means for human beings with long‐term illness to be in the movement between suffering and health and their motives and resources to promote and maintain health.

**Methodology and Method:**

This study has an inductive qualitative research design with a hermeneutic approach. The data collection was carried out via in‐depth interviews with seven people with long‐term illnesses. The interviews were analysed inductively using reflexive thematic analysis.

**Results:**

Three main themes emerged in the thematic analysis: *Moving between letting go and holding on*, *a changed attitude towards health* and *trusting life to carry you*. The results demonstrate that for a human being with a long‐term illness, the movement between suffering and health is the reality in which they live and as a movement between giving up and holding on, between grief and gratitude, and between vulnerability and nearness.

**Conclusion:**

When human beings face suffering in the form of long‐term illnesses, they can experience health in the ways they choose to act, relate and view their value as a human being, without any requirement for performance. The task of healthcare is to recognise the resource that people themselves possess. Healthcare professionals need a dialectical view of health and suffering to provide care that not only aims to eradicate disease but also to see what health is in times of illness.

## 1 Introduction

When faced with a long‐term illness the fear of losing our health sets in, which changes the way in which we perceive our own health and what it means to us. What used to be an unquestioned line between illness and health is fading. Previous research indicates that health can coexist with illness and suffering [1, 2]. Suffering can even reinforce man's search for meaning, which in turn has implications for health [3, 4]. Research related to specific diseases show that people suffering from long‐term illness experience health even if they are ill. For example, Harrop et al. [5], who studied people suffering from advanced lung cancer, highlight in their findings that one way of coping with life after receiving the diagnosis is trying to understand and manage the illness, continuing with daily routines, and confronting the future. Olano‐Lizarraga et al.'s [6] study on personal experiences of living with chronic heart failure indicates that living with the disease affects the whole human being, and that the ability to seek the positive and to use internal resources and external support are key strategies for managing life.

The question of health in times of illness is largely about how health is characterised. A view on health centred on the body and abilities usually implies that health cannot be achieved when the human being suffers from disease. If health is characterised as well‐being, then human beings can have health despite illness [7]. In a study on women with Parkinson's disease, Olsson and Nilsson [8] convey that health is about feeling well, and that even in challenging situations it is possible to experience health. Health was described as the possibility of feeling harmony, being in a community, and a possibility of moving on despite the illness.

Health is also related to a sense of meaning and individuals who experience meaning in their lives are better able to handle periods of illness [9, 10, 11]. Zhang et al.'s [12] study on chronically ill older people shows that health can be experienced as a dialectical movement between accepting the inevitable and a willingness to deal with the challenges of the illness. According to Reb [13], the experience of meaning is tied to hope for women with ovarian cancer. Support and an awareness of control can move women from a sense of being doomed to one of hope and health.

Previous research reveals that human beings can have health despite chronic or severe illness. Earlier research has to a great extent focused on health related to specific diseases grounded in coping strategies or experiences of meaning in life [14]. This hermeneutical caring science study highlights a deeper understanding of health as a movement between suffering and health and human beings' motives and resources for promoting and preserving health during long‐term illness.

### 1.1 Theoretical Standpoints

The theoretical standpoint of this study originates in caring science and Eriksson's multidimensional health theory [15, 16, 17, 18]. According to Eriksson, health and suffering are integrated parts of human life, and human life is to strive to reach one's potential by moving between suffering and health [16, 17, 18]. Health is thereby more than the absence of illness and cannot be reduced merely to a lack of illness because a person can feel healthy despite being ill. Health is also understood as a dimension of becoming or growing towards health which means an inner striving for well‐being, harmony, meaning in life, and wholeness [15]. In the deeper dimensions of health, human beings can be aware of their inner resources, strive towards light, shape hope, and find deeper motives for promoting and preserving health. Through facing suffering, human beings can learn to endure illness, and suffering can become a positive resource, a force in life, joy, and desire for life [18].

### 1.2 Aim

This study aims to gain a deeper understanding of health, what it means for human beings with long‐term illness to be in the movement between suffering and health and their motives and resources for promoting and preserving health.

## 2 Methods

### 2.1 Methodology

This study has an inductive qualitative research design with an open hermeneutic approach based on Gadamer's hermeneutic philosophy. According to Gadamer [19], understanding involves grasping the meaning of the whole through an ongoing dialectical movement between the whole and its parts. To identify patterns of meaning thematic analysis according to Clarke and Braun [20] has been used. Thematic analysis is a method, Clarke and Braun [20] points out that the researchers have to define the methodology and carry out the analysis in unity with chosen methodology. The study is thereby guided by Gadamer's [19] view of hermeneutical methodology and epistemology and the theoretical framework of Eriksson's view on health [18].

The pre‐understanding for this study is based on understanding off previous research findings of renunciation and health experienced by people with long‐term illnesses [21]. The results showed themes of recognising one's vulnerability, allowing oneself to grieve, courage to face life's questions, to see the light despite the darkness and to let go but not give up. In accordance with the hermeneutic methodology, the findings of the previous study raised an interest to deepen the understanding of the movement between suffering and health, raised new questions and a will to carry out a secondary data analysis of the collected data. The pre‐understanding also includes the review of previous research carried out specifically for this study as well as the theoretical framework of the study.

### 2.2 Data Collection

The data collection was carried out in 2018 via in‐depth interviews [22] with people with long‐term illnesses. By using secondary data analysis, new research questions were asked to the material to gain a deeper understanding of health and illness. Secondary data analysis can give new insights of the interviews and extend the understanding. The authors have collected the data themselves and therefore have an understanding of the context in which the interviews were originally conducted, and therefore the epistemology, methodology, and ethical stance of the previous project and the current study are in line [23]. According to Bishop [24], the process of a secondary analysis is very similar to primary data analysis. The movement between the research question and the data is the same, as is the movement back and forth in the material as a whole.

Participants were selected through a combination of appropriate selection and snowball sampling [25, 26]. Participants in the study were individuals living at home with a long‐term illness that had significantly affected their living situation for at least 1 year. Seven people participated in the interviews, two men and five women. The age of the participants varied between 30 and 75 years and the time passed since the illness appeared or was diagnosed varied between 1 and 52 years. To gain a more general understanding, participants with different illnesses were included in the study, although the common denominator was that they were long term (multiple sclerosis, cancer, stiff person syndrome, renal failure, neurological disease, severe fatigue disorder and type 1 diabetes). In line with the hermeneutic approach of the study, the questions and the approach were slightly refined after each interview in order to obtain answers that corresponded to the purpose of the study. The interviews focused on two themes, renunciation and health. Follow‐up questions were asked to develop and deepen the conversation. The secondary data analysis conducted for this study focused on the data describing their perception of health and suffering and their motives and resources for promoting and preserving health. The interviews were conducted in Finland in the participants' homes or at their work, in a place and environment where they felt comfortable and safe. The interviews lasted approximately 1 h each. Recorded interviews were transcribed into a total of 138 pages of text. The first author conducted the interviews.

### 2.3 Data Analysis

The data were analysed inductively using reflexive thematic analysis according to Braun and Clarke [20]. The analysis began with a first reading to allow the researchers to become more aware of the whole of the interviews. The next step was to code the data in a reading process that involved both a literal and conceptual interpretation of the material. The focus of the coding was to capture passages that had relevance to the research objective [27]. A total of 187 codes were identified. In step three, the codes were written down on notecards and sorted into patterns of meaning to form subthemes and in turn, were read and interpreted to result in themes. Twenty‐five subthemes and nine themes were identified (see Table 1). As part of the hermeneutic interpretive spiral, in step four the authors returned to the codes and reread them to see if the themes formulated worked in relation to the codes that had been abstracted. The entire data set was then reread, and a thematic map was created. In step five, the themes that were found to have many similar characteristics were merged, brought together, and finally formulated into three main themes. The three main themes show the overall story that the analysis is telling (see Table 1). In step six, the three main themes were defined and described with text to gain a deeper and more concise understanding of the themes and their interrelationships. Quotes were used to illuminate and reinforce the analysis. The analysis included interpretation based on the hermeneutic approach of the study. To ensure credibility and trustworthiness in the analysis and interpretation, both authors participated in the analysis.

**TABLE 1:** Theme development.

Subthemes	Themes	Main themes
Open to a more permissive life Let go of what doesn't work Focus on the essentials of life	Holding on to the good in life	Moving between letting go and holding on
Giving up old habits breeds creativity Challenge previous thought structures	Tread new paths in a pathless landscape	
Let go of the need for control Surrender but not resign Let go of what hinders health Allow oneself to grieve	Let go but don't give up	
Choosing to see the positives Let hope live Not letting the darkness take over	See the light despite the darkness	A changed attitude towards life
Dare to ask the difficult questions Emphasise core values	Courage to face the questions of life	
Gratitude for what you have Illness is no obstacle to happiness Presence in the present	Finding health in the present	
Don't give up the will to live Let go of bitterness and shame	A desire to live authentically	Trusting life to carry you
Affirm one's loneliness Give up independence Accept dependence on others	Living as vulnerable	
Live with uncertainty It is enough to just be Embrace one's limits	Resting in being	

## 3 Results

The results are presented based on the three main themes that emerged in the thematic analysis: *moving between letting go and holding on*, *a changed attitude towards life* and *trusting life to carry you*. Each main theme is described based on themes and subthemes and illustrated with descriptive quotes.

### 3.1 Moving Between Letting Go and Holding on

The first theme shows that health in times of illness is about holding on to what brings health and letting go of what hinders health. This was explained as becoming present at different stages of the illness. In the early phase, holding on can be about smaller things such as sticking to routines that provide stability in everyday life or giving up certain habits that are no longer possible, but also holding on to what one considers valuable in life and letting go of more trivial things that were previously allowed to take place. The choice to hold on to something or to let go is both easy and difficult. In some respects, letting go and embracing new ways is obvious, but sometimes it is a struggle.

I was only forty when I needed a walker, it was terrible. I cried the first time I walked with my walker. (P1)

When certain ways of doing things no longer work because of illness, creativity and persistence to find new ways of doing things can be born. Even when old things that one was used to doing had to be completely abandoned, wholly new things were discovered. Sometimes the illness became like a new ‘job’, something to put energy into and focus on, a problem to solve.

Many different types of emotions were described in relation to what human beings must give up because of illness. Anxiety, anger, and disappointment were some of the difficult ones, but also positive feelings of relief and liberation were mentioned. The emotional, rational and practical significance of having to let go of something that was previously taken for granted can be understood as an inner movement that ebbs and flows. The rational and practical sometimes speak a very clear language while the emotions have their voice linked to different forms of values. This sometimes causes conflicts with which one must struggle. One participant said: ‘Either you lie down and give up, or you fight on with the premises you have’ (P4).

The results highlight a need to grieve, to let grief take place, and to give oneself permission to be angry, otherwise there is a risk of becoming bitter. Facing illness raises issues of fairness and so there is an increased risk of bitterness. Different ways of dealing with these feelings were expressed. Examples of ways to manage bitterness were praying to avoid it or focusing on what they have rather than what they are missing.

I received a prayer within me: I must not become bitter because then I will spoil life for myself and others. My faith is important to me. It gives me light and hope. I have inner security and I know that I am valuable even when I am ill. (P1)

Loss and grief associated with illness awaken a need to hold on to what is valuable and core values work as a guide in navigating a new and uncertain situation. While there is nothing positive about the illness, it can reveal core values and help the individual to focus on what is most important in life. One participant said: ‘Finding out what is meaningful to you and what you are meant to do is not easy, but it is valuable’ (P5).

Other values that promoted health were the ability to be close to family and friends, and concern for the well‐being of family members. Still being able to contribute to others seems to be a core value for people with long‐term conditions, not always being the recipient of, but being able to give care to others.

### 3.2 A Changed Attitude Towards Life

The findings underscore that being diagnosed with a serious or chronic illness causes various forms of suffering in the form of reduced mobility, disabilities, pain, depression, anxiety and existential questions. Experiencing suffering from a long‐term illness implies being faced with a life‐changing reality. The familiar and known life was forever changed for oneself and one's loved ones. How to navigate life through suffering can therefore be a great concern and burden but also an active choice to focus on the good and the positive rather than the difficult and burdensome nature of the illness. Having a thankful and grateful attitude was seen as beneficial to health. Ultimately, it is about focusing on the things one has that help one to deal with what one does not have.

I realize now that my attitude has changed from having to be in a wheelchair to what a privilege it is to be able to move around more freely with the wheelchair. It has changed the way I think. (P3)

A core attitude towards health was that bodily function and the absence of illness are not prerequisites for health. One participant said: ‘You can experience health in a sick body, and you can feel very sick in a healthy body’ (P1). The harsh reality seems to help the individual to focus on what is important and to change the attitude to a more grateful one, where the present and what one has is of more value than an uncertain future and what one may lose due to illness.

Fear of the unknown future also brings out a range of emotions and concerns. Before an illness one can feel as though one is in control of one's life, make plans and dream about what life would be like in the future, but with long‐term illness one never knows what the future will be like. When faced with a long‐term illness and uncertainties about the future, a change in attitude becomes essential. Enjoying the presence, each moment, seeing something positive every day, and delight in what one has are the best ways to experience health when facing an unclear future. Changing one's attitude towards life and being more present is sometimes difficult, but children are seen as good helpers in staying in the present and enjoying small things.

### 3.3 Trusting Life to Carry You

The third theme of this study shows that when individuals no longer have the ability or strength to be in control of their lives they put their trust in life itself to carry them. In this case, life stands for something that represents a form of trust for the human being; sometimes illustrated in concrete terms as trust in other people or healthcare, or in something more abstract such as God or the universe.

Performance is not necessary for the experience of health. As abilities and choices are taken away the possibility and ability to just be as a way of experiencing health becomes essential. What could be described as the essence of health for people with a long‐term illness is just being human. It is enough to be human and being human is understood as not requiring performance. One participant said: ‘I just have to be me. I have bad days, but I have learned that I don't have to do anything, I just have to be me’ (P4). While the illness deprives humans of many things, it also allows them to live authentically, without the safety of credentials and achievements.

A sense of being utterly alone in the suffering and illness simultaneously led to an opening up of a deep communion with others. Sharing the experience with others in a similar situation provided a sense of community and support. When many of the things that one thought would be there in the future were taken away by the illness, there is also a risk of feeling bare, exposed and cut off from others. At the same time, vulnerability, nakedness, and loneliness enable closeness to others and give a sense of health despite illness.

What I found most difficult was this helplessness that came into my life. I had always managed on my own. Now I was suddenly dependent on others. (P4)

Trust was implied as necessary to experience health when feeling vulnerable. The value of trusting other people and someone or something bigger to carry them when they are unable to carry themselves emerged in the results. When human beings trust, they put their faith in the other and believe that just being is sufficient for them.

## 4 Discussion

The results demonstrate that for human beings with long‐term illness, the movement between suffering and health is the reality in which they live. This movement is reflected in all the themes. The participants describe a movement between giving up and holding on, between grief and gratitude, and between vulnerability and nearness. This highlights how healthcare professions can understand health in relation to illness. Health is not static, and it cannot be captured or understood other than by describing the different patterns of movement. This is consistent with a caring science dialectic understanding of health [5, 18].

On a quite concrete note, the participants expressed a movement between what they should hold on to and what they need to let go of in order to experience health. This is where creativity and the ability to choose new paths come to the fore. Trying to solve the problems that arise with creativity and persistence can be a way of experiencing well‐being; what was an obstacle becomes a challenge which, if new ways are found, can lead to a sense of fulfilment. This is also shown in a study by Hedlund et al. [28], where persons with serious illnesses conveyed that they had to find new ways to solve the challenges posed by the illness, find new ways of doing things or find other interests when what they were previously interested in was no longer possible.

The second movement that the findings point to is the change in attitudes towards life. What was previously prioritised as important takes a secondary position as deeper values are exposed by the suffering [11, 12]. Living in the moment and being grateful for what is highlighted as a way to meet the change in life that the illness entails. The movement between health and suffering is made clear in the loss of control over one's life, not knowing what will happen. This movement can be seen in a time perspective, between what has been and what is to come. In a study focusing on incurable cancer patients coping in hospice care, Viitala et al. [29] found that positive thinking and focusing on life were more important to patients than thinking about their own impending death. This finding is interesting as life itself also seems to be the focus for the participants in our study. Perhaps this movement is a sign of life and that is why it is important to recognise and promote movement for human beings in all phases of life.

The results show that this movement can be understood as both a struggle and an attitude, and as surrender to what it in its deepest meaning means to be human, to be naked and vulnerable. Vulnerability is often described as something negative for human beings to be protected from or against. The findings of this study reveal, however, that vulnerability can be seen as strength in human life, as something that makes it possible for individuals to live their lives to the fullest, despite the loss of strength and abilities. Zagorac and Stamenković Tadić [30] write about how contemporary research often focuses on vulnerability as a weakness, something to be alleviated. They take us back to ancient Greek philosophy where vulnerability was seen as a power for change. Zagorac and Stamenković Tadić [30] put forth the importance of seeing the potential for transformation in human beings through vulnerability.

Becoming ill can mean that a person's self‐image changes, as the achievements that were previously part of their being are no longer possible. Svenaeus [31] describes it as a feeling of homelessness that overcomes the human being in the face of suffering and where the struggle is to remain at home in the face of the futility of suffering. According to Kristensson Uggla [32], what makes us human is the ability to de‐centre the self. To allow the ‘inner’ to meet the ‘outer’, to see life as a gift and not as an achievement. This is where healthcare should meet the suffering human being, where their ‘inner’ meets their ‘outer’. According to Eriksson's [18] theory of multidimensional health, the innermost level of health is ‘becoming’. It can be understood as the ‘inside’ of the human being meeting the ‘outside’ (the caregiver, another human being, an abstract other) and coming into being [33, 34]. A reorientation towards what it means to be human, towards the essence of being human, can help people to experience health in spite of illness and limited abilities.

## 5 Strengths and Limitations

One strength of the present study lies in that it gives people with different forms of illness, different ages and genders a voice and that the results demonstrate a deeper understanding of health in relation to illness on an ontological level and not just in relation to a given disease. Another strength is that the first author interviewed and collected all the data herself, which means that the trustworthiness of the analysis and interpretation can be considered strong. The small number of participants can be seen as a limitation; more informants would have increased the transferability of the findings.

## 6 Conclusion

The role of healthcare is to alleviate patient suffering and promote health. The results of the study show that these are not two different tasks, but health and suffering are integrated and mutually dependent, which means that the caregiver must always relate to both aspects of care. When human beings face suffering in the form of long‐term illnesses, they can experience health through how they choose to act, relate and view their value as a human being, without any requirement for performance. The task of healthcare is to recognise the resources that people themselves possess.

To alleviate suffering will always be the core of caring, but this study demonstrates that there is, and can be, health in times of illness. Caring is not meant to eradicate the vulnerability that suffering brings, as it seems to have the ability to bring human beings closer to their core and can conversely bring health despite illness. More research is needed to develop the view on health related to illness. Healthcare professionals need a dialectical view of health and suffering to provide care that not only aims to eradicate disease but also to see what health is in times of illness.

## Author Contributions

M.K. and C.A.K. conceptualised and designed the study. M.K. was responsible for data collection. Both M.K. and C.A.K. conducted the data analysis. M.K. drafted the manuscript under the supervision of C.A.K. All authors reviewed and approved the final version of the manuscript.

## Ethics Statement

This study has followed the guidelines for responsible conduct of research and procedures for handling allegations of misconduct in Finland published by the Finnish National Board on Research Integrity. All interviews were conducted respectfully, and everyone's identity was protected. An ethical statement was requested and obtained from the research ethics committee at Åbo Akademi University, Finland, in the spring of 2018. All participants received information about the study and what participation in the interview entailed and signed a consent form to participate in it. A risk assessment was carried out and participants were also offered support if the interview raised feelings that they needed help with. All data collected was handled according to the Åbo Akademi University's policy on research data management. The anonymity of the results was ensured by giving each participant a number code (P1‐P7).

## Conflicts of Interest

The authors declare no conflicts of interest.

## Data Availability

The data that support the findings of this study are available on request from the corresponding author. The data are not publicly available due to privacy or ethical restrictions.
